# Immune Checkpoint Receptors Tim-3 and PD-1 Regulate Monocyte and T Lymphocyte Function in Septic Patients

**DOI:** 10.1155/2018/1632902

**Published:** 2018-11-22

**Authors:** Qi Xia, Li Wei, Yuntao Zhang, Jifang Sheng, Wei Wu, Yi Zhang

**Affiliations:** ^1^State Key Laboratory for Diagnosis and Treatment of Infectious Diseases, The First Affiliated Hospital, College of Medicine, Zhejiang University, Hangzhou 310003, China; ^2^Department of Intensive Care Medicine, The First Affiliated Hospital, College of Medicine, Zhejiang University, Hangzhou 310003, China; ^3^Department of Laboratory Medicine, The First Affiliated Hospital, College of Medicine, Zhejiang University, Hangzhou 310003, China; ^4^Key Laboratory of Clinical In Vitro Diagnostic Techniques of Zhejiang Province, Hangzhou 310003, China

## Abstract

We aim to investigate the effects of Tim-3 and programmed cell death-1 (PD-1) on the monocytes and T lymphocytes in septic patients. Expression of Tim-3 and PD-1 on the CD3, CD4, and CD8 lymphocytes and monocytes was determined using flow cytometry. CBA technique was utilized to determine the expression of cytokines in the lymphocyte supernatant in addition to the IL-10 and TNF-*α* positivity in monocytes in the presence of Tim-3 and/or PD-1 receptor blockade. Compared with the normal control, significant elevation was observed in the expression of PD-1 on CD3 (*P* = 0.004), CD4, and CD8 monocytes. Blockade of the Tim-3 signaling pathway contributed to the significant elevation of IL-10 and TNF-*α* in the supernatant of T lymphocytes in the septic patients, while the PD-1 signaling pathway blockade only triggered the obvious elevation of TNF-*α* in the T lymphocytes. Blockade of Tim-3 and PD-1 induced the positivity of IL-10- and TNF-*α*-expressing cells in the peripheral monocytes. Significant changes were noticed in the Tim-3 and PD-1 in the T lymphocytes and monocytes. Blockade of Tim-3 and PD-1 contributed to the function of lymphocytes and monocytes. In the septic process, Tim-3 and PD-1 played crucial roles in the immune response of T lymphocytes and monocytes.

## 1. Introduction

Sepsis, a life-threatening organ dysfunction caused by a dysregulated host response to infection [1], is still a challenge as it is one of the leading causes for mortality in ICU despite the advances in the diagnosis and treatment of such disease [2–5]. To date, extensive studies have been performed to investigate the pathogenesis of sepsis which are aimed at facilitating the diagnosis and treatment, as well as improving the survival rates. Among these studies, the immunological function of human body is considered to play crucial roles in the sepsis [1, 4–6]. Besides, even the survivors after sepsis also showed severe immune suppression in a long time [7]. Thus, the immunosuppression in septic patients, especially these with functional defect of T lymphocytes and monocytes, has been considered as a new hot topic in the field of sepsis [8, 9].

T cell immunoglobulin- and mucin-domain-containing molecule-3 (Tim-3) and programmed death-1 (PD-1) are two important regulatory molecules in cell-mediated immunity that are expressed in various immune cells, including T lymphocytes and monocytes [10, 11]. In the previous description, Tim-3 and PD-1 have been reported to be closely involved in the pathogenesis of tumor, autoimmune diseases, and chronic viral diseases [11–17]. These two factors also mediated the functional failure of T lymphocytes, and several studies have been carried out to determine their roles in sepsis; however, the exact mechanisms are still not well defined [18].

In this study, we aim to investigate the expression of Tim-3 and PD-1 in T lymphocytes and monocytes of the peripheral blood in septic patients. Besides, their roles in mediating immune response were determined.

## 2. Material and Methods

### 2.1. Subjects

Twenty-three septic patients admitted in the ICU of the First Affiliated Hospital of Zhejiang University from January 2017 to July 2017 were included in this study. Diagnosis of sepsis was based on the Third International Consensus Definitions for Sepsis and Septic Shock (Sepsis-3). Besides, 20 healthy individuals recruited from the Physical Examination Center of the First Affiliated Hospital of Zhejiang University during the same time period served as normal control. The exclusion criteria were as follows: those aged < 18 years, with a history of autoimmune diseases, a recent infection within 6 months, cancer, and pregnancy, as well as a history of human immunodeficiency virus (HIV) infection. Blood samples were collected within 24 hrs after confirmation of sepsis. The demographic and clinical characteristics of subjects are summarized in Table 1. Written informed consent was obtained from each subject. The study protocols were approved by the Ethics Committee of Zhejiang University.

### 2.2. Flow Cytometry

**Figure 1:**
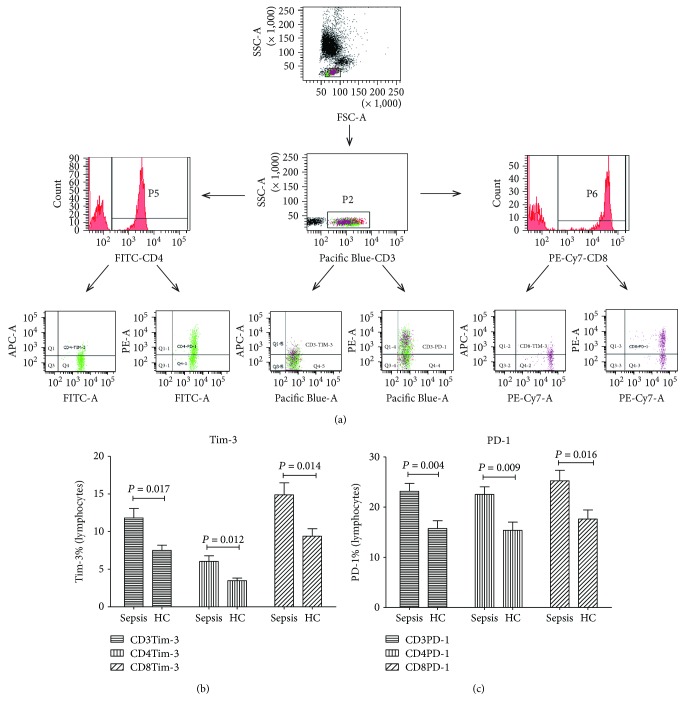
Tim-3 and PD-1 expression in 23 sepsis patients and 20 HC by flow cytometry. (a) Flow cytometry gating strategy for the determination of Tim-3(+) and PD-1(+) on T cells. (b) The percentages of CD3+Tim-3+, CD3+CD4+Tim-3+, and CD3+CD8+Tim-3+ T cells. (c) The percentages of CD3+PD-1+, CD3+CD4+PD-1+, and CD3+CD8+PD-1+ T cells. Statistical analysis was performed by the Mann–Whitney test.

To determine the frequency of distinct leukocyte subpopulations, heparinized blood was lysed using TQ-Prep (Beckman Coulter), followed by staining with antibodies against CD3-Pacific Blue, CD4-FITC, CD8-PECy7, TIM-3-APC, PD-1-PE (BD Bioscience), as well as evaluating using flow cytometry (Canto-II, BD). Gating strategy of different immune cell populations was shown in Figure 1. The frequencies of granulocytes, lymphocytes, and monocytes within the leukocyte population were determined based on their FSC-SSC profile. Isotype-matched immunoglobulins served as control.

### 2.3. Measurement of Plasma Cytokine Levels

The concentrations of serum IFN-*γ*, TNF-*α*, IL-2, IL-4, IL-6, and IL-10, were determined from each subject using a cytometric bead array (CBA) according to the manufacturer's instructions. All the tests were performed at least in triplicate.

### 2.4. Intracelluar Cytokine Assays

Peripheral blood mononuclear cells (PBMCs) from subjects were stimulated with Dynabeads Human T-Activator CD3/CD28 (Dynal, Invitrogen) for 6 hrs. CD3/28 beads served as the stimulator for the lymphocytes in PBMCs, and the supernatant was utilized to detect the lymphatic function. LPS served as the stimulator for monocytes in PBMCs and was used to detect their function. Then, monensin (1.7 *μ*g/ml, Sigma-Aldrich) was added to inhibit cytokine secretion. The cells were stained with anti-CD3 or anti-CD14 for 20 min at 4°C, followed by fixation and permeabilization. The cells were then stained with anti-TNF-*α*-PE, anti-IL-10-PE, or isotype control for 30 min at room temperature, followed by flow cytometry.

### 2.5. Statistical Analysis

Data were expressed as mean ± standard deviation (SD) or number (percentage). The difference of independent data between two groups was analyzed by the Mann–Whitney nonparametric test. Chi-square test was utilized for the comparison of gender structure between the patients and normal control. One-way ANOVA test was used for the comparison between the age of patients and normal control. A two-sided *P* value of <0.05 was considered statistically significant. All the analyses were performed using SPSS 16.0 for Windows (SPSS, Chicago, IL).

## 3. Results

### 3.1. Number of White Blood Cell, Lymphocytes, and Monocytes in the Peripheral Blood in Septic Patients

Table 1 showed the number of white blood cell, lymphocytes, and monocyte in the peripheral blood in septic patients. Compared with the normal control, the number of white blood cell showed significant elevation in the septic patients (*P* < 0.001), whereas the absolute number and percentage of lymphocytes showed significant decrease in the septic cases (*P* < 0.001). No statistical differences were noticed in the monocytes in the septic patients compared with that of the normal control (*P* > 0.05).

### 3.2. Expression of Tim-3 on the T Lymphocytes and Monocytes in Septic Patients

**Figure 2:**
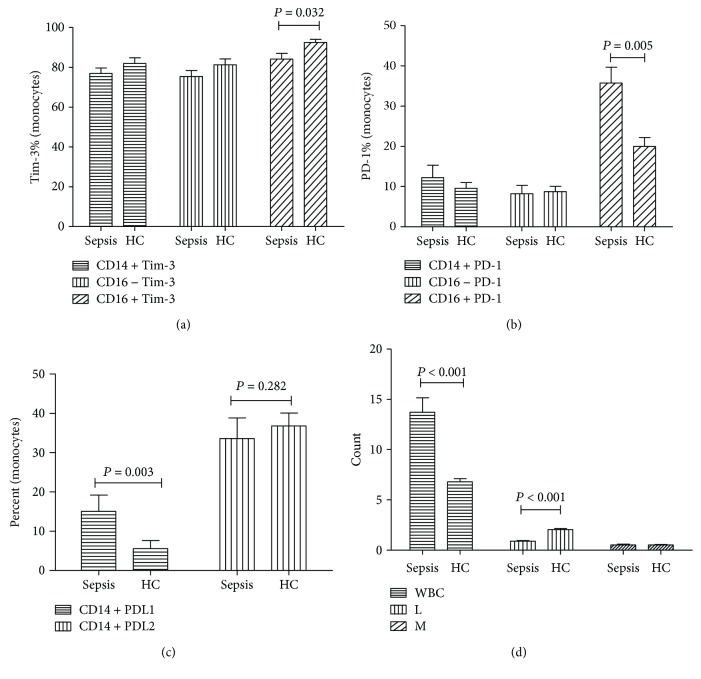
(a) The percentages of CD14+Tim-3+, CD14+CD16−Tim-3+, and CD14+CD16+Tim-3+ on monocytes. (b) The percentages of CD14+PD-1+, CD14+CD16−PD-1+, and CD14+CD16+PD-1+ on monocytes. (c) Percentage of PDL1 and PDL2 on monocytes. (d) Count of white blood cells, lymphocytes, and monocytes. Statistical analysis was performed by the Mann–Whitney test.

Flow cytometry indicated that significant increase was noticed in the expression of Tim-3 on the CD3+ (*P* = 0.017), CD3+CD4+ (*P* = 0.012), and CD3+CD8+ T lymphocytes (*P* = 0.014) in the septic patients compared with that of the control group (Figure 1(b)). Compared to the lymphocytes, the expression of Tim-3 on the CD14+16+ monocytes showed significant decrease (*P* = 0.032). The expression of Tim-3 on the CD14+ monocytes and CD14+16− monocyte subpopulation showed no statistical differences compared with those of the control group (*P* > 0.05, Figure 2(a)).

### 3.3. Expression of PD-1, PD-L1, and PD-L2 on the Expression of T Lymphocytes and Monocytes in Septic Patients

The expression of PD-1 on the CD3+ lymphocytes (*P* = 0.004), CD3+CD4+ lymphocytes (*P* = 0.009), and CD3+CD8+ lymphocytes (*P* = 0.016) showed significant increase compared to the control (Figure 1(c)). Besides, the expression of PD-1 on the CD14+16+ monocytes showed significant increase compared to that of the normal control (*P* = 0.005, Figure 2(b)). For the PD-1-related ligands, the expression of PD-L1 showed significant increase in the monocytes in septic patients compared with that of the normal control (*P* = 0.003); however, no statistical differences were noticed in the expression of PD-L2 in the monocytes between the two groups (*P* > 0.05, Figure 2(c)).

### 3.4. Blockade of the TIM-3 and PD-1 Signaling Pathway Contributed to T Lymphocyte Expression of Cytokines

**Figure 3:**
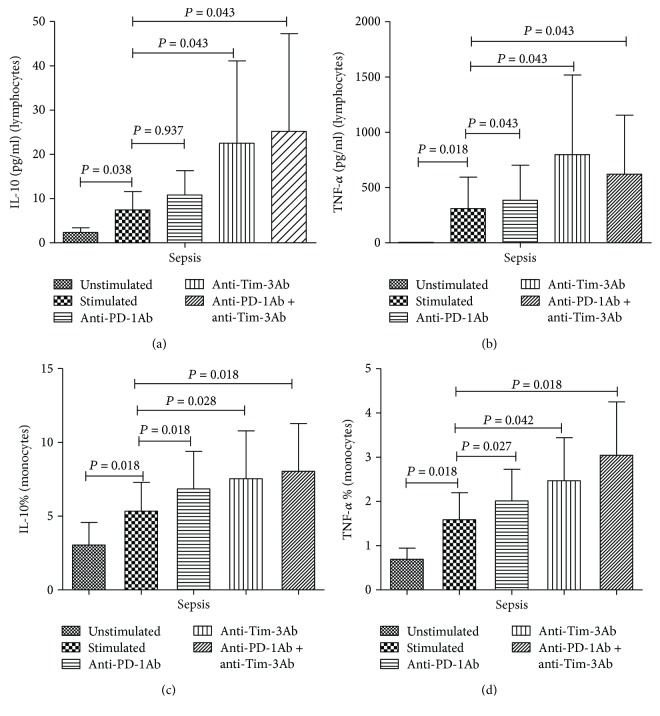
Effects of the Tim-3 and PD-1 signaling pathway on the cytokines in the T lymphocytes and monocytes. (a) Expression of IL-10 in T lymphocytes after Tim-3 and PD-1 blockade. (b) Expression of TNF-*α* in T lymphocytes after Tim-3 and PD-1 blockade. (c) Proportion of IL-10-expressing monocytes after Tim-3 and PD-1 blockade. (d) Proportion of TNF-*α*-expressing monocytes after Tim-3 and PD-1 blockade. The data were analyzed by the Mann–Whitney test.

We determined the expression of IL-2, IL-4, IL-6, IL-10, IFN-*γ*, and TNF-*α* in the supernatants in the following sets: subject to no simulation, bead, bead plus PD-1 antibody blockade, bead plus Tim-3 antibody blockade, bead plus PD-1 antibody, and Tim-3 antibody blockade, respectively. Tim-3 antibody blocking may induce significant increase of TNF-*α* and IL-10 in the T lymphocytes after stimulating with bead. However, in the presence of PD-1 antibody blockade, only the serum TNF-*α* showed elevation and the other cytokines showed no changes in secretion (Figures 3(a) and 3(b)).

### 3.5. Blockade of the Tim-3 and PD-1 Signaling Pathway Triggered Increase of Monocytes Secreting IL-10 and TNF-*α* in Septic Patients

In this section, we determined the expression of IL-10- and TNF-expressing monocytes in the absence of stimulation, in the presence of LPS stimulation, LPS stimulation and PD-1 antibody blockade, LPS stimulation and Tim-3 antibody blockade, and LPS stimulation and PD-1 and Tim-3 antibody blockade. Our data showed that the proportion of IL-10- and TNF-*α*-secreting monocytes increases in the presence of LPS stimulation after PD-1 and Tim-3 antibody blockade (Figures 3(c) and 3(d)).

### 3.6. Measurement of Serum Cytokines

**Figure 4:**
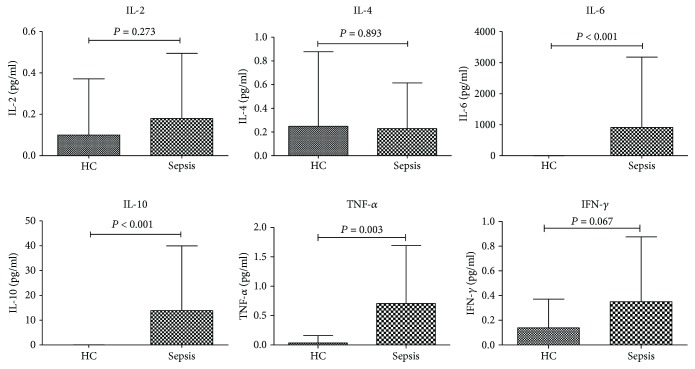
Content of serum cytokines in septic patients and normal individuals.

In this section, we determined the IL-2, IL-4, IL-6, IL-10, TNF-*α*, and IFN-*γ* in the peripheral blood in patients and normal control. Compared with the normal control, the serum IL-6 (*P* < 0.001), IL-10 (*P* < 0.001), and TNF-*α* (*P* = 0.003) showed significant increase in the septic patients (Figure 4).

## 4. Discussion

Immune functional imbalance plays a pivotal role in the T lymphocyte and monocyte dysfunction in septic patients [5]. Moreover, it is the major cause for the opportunistic infection and secondary infection in these patients. In this study, we found that the expression of Tim-3 and PD-1 showed significant changes in the T lymphocytes and monocytes in the peripheral blood in septic patients. Besides, blockade of Tim-3 and PD-1 signaling pathways contributed to the recovery of cytokine secretion.

The regulation of Tim-3 was modulated by environmental factors, and the upregulation of Tim-3 expression was associated with the proliferation, apoptosis, and functional failure of the lymphocytes [17, 19, 20]. Our previous studies showed that the Tim-3 expression on peripheral T cell subsets was correlated with disease progression in hepatitis B infection and blockade of Tim-3 signaling could restore the virus-specific CD8+ T cell response [13, 14]. In vitro experiments showed that Tim-3 antibody blockade involved in regulating the secretion of TNF-*α* and IFN-*γ*. In this study, we found that the expression of Tim-3 in the peripheral blood in septic patients showed significant elevation and the number of lymphocyte count showed significant decrease in these patients compared with that of the normal control. This, together with our previous description [13], indicated that blockade of the Tim-3 signaling pathway promoted the proliferation of T lymphocytes. On this basis, we considered that the high expression of Tim-3 on the lymphocytes may be associated with the apoptosis of the T lymphocytes, which may further trigger in the decrease of lymphocytes.

According to our previous description, the decreased Tim-3 expression was associated with functional abnormalities of monocytes in decompensated cirrhosis without overt bacterial infection. Besides, the endotoxin may induce the downregulation of Tim-3 on the monocytes. Zhang et al. reported that the expression of Tim-3 on the monocytes was downregulated after the activation of Toll-like receptors in the monocytes [21], which may be related to the infection, elevation of endotoxin, and massive activation of Toll-like receptors [21]. The monocytes could be divided into various subsets according to the expression of CD16. The CD14+CD16+ monocytes were closely related to the immune response, and its number showed significant increase in the septic patients [22, 23]. Our data showed that the decrease of Tim-3 was only in the CD14+CD16+ monocytes, while no significant changes were noticed in the expression of CD14^+^CD16^−^ monocytes. On this basis, we speculated that Tim-3 was mainly involved in the regulation of CD16+ monocytes during the pathogenesis of sepsis.

PD-1 is mainly responsible for the maintenance of immune balance through inhibiting the aggressive immune responses. PD-1 could induce T cell exhaustion and downregulate the proliferation of CD4+ and CD8+ T lymphocytes as well as the cytokines. Also, the proliferation of T lymphocytes showed significant increase after PD-1 blockade [24]. In animal models, PD-1 antibody could attenuate the decrease of white blood cell count in septic patients and inhibit the apoptosis of lymphocytes, which then improved the survival rates [9, 25, 26]. PD-L1 and PD-L2 are the two forms of PD-1 receptors, which show difference in the structure and function. Among these two forms, PD-L1 played important roles. Our data showed the PD-1 expression on the T lymphocytes, and CD14+CD16+ monocytes showed significant increase in the septic patients together with the PD-L1 on the CD14+CD16+ monocytes compared to those of the normal control. Therefore, it was reasonable to speculate that PD-1 may involve in the regulation of T lymphocytes and monocytes in septic patients, especially in the regulation of CD16+ monocytes that were closely related to the inflammation.

In this study, we firstly investigated the secretion of T lymphocytes and monocytes in septic patients through Tim-3 and PD-1 antibody blockade in vitro. Our data showed that the secretion of IL-10 and TNF-*α* in the T lymphocytes increased after Tim-3 antibody blockade, while PD-1 blockade enhanced the secretion of TNF-*α* by T lymphocytes. The other cytokines showed no statistical differences after blockade. After Tim-3 and PD-1 antibody blockade, the IL-10- and TNF-*α*-secreting monocytes showed increase in number in the peripheral blood in septic patients. This implied that Tim-3 and PD-1 blockade contributed to the recovery of secretion function of lymphocytes and monocytes in different ways. Interestingly, simultaneous blockade of these two signaling pathways caused no significant increase in the secretion function compared with single blockade of Tim-3 or PD-1. We hypothesized that there was redundant overlap between Tim-3 and PD-1 signaling pathways. Therefore, simultaneous blockade of both pathways triggered no significant net efficacy than blockade of either alone. In the future, further studies are required to investigate the exact potential mechanisms.

## 5. Conclusions

In conclusion, Tim-3 and PD-1 played crucial roles in regulating the immune responses of monocytes and T lymphocytes in septic patients. In the near future, these two molecules may serve as potential targets for the immunotherapy of sepsis.

## Figures and Tables

**Table 1:** The demographic and clinical characteristics of subjects.

Variables	Patients with sepsis (*n* = 23)	Healthy controls (*n* = 20)	*P*
Age (year)	57.30 ± 10.18	54.40 ± 10.26	0.358^#^
Male (%)	69.57%	65.00%	0.75^†^
WBC	13.86 ± 6.58	5.74 ± 1.00	<0.001^Δ^
*L* (absolute lymphocyte count)	0.90 ± 0.32	1.90 ± 0.38	<0.001^Δ^
Lymphocyte (%)	7.40 ± 2.90	30.21 ± 4.34	<0.001^Δ^
Monocytes (%)	0.54 ± 0.30	0.50 ± 0.23	0.88^Δ^
APACHE II	21.94 ± 8.09	—	—
Length of ICU stay	18.78 ± 17.27	—	—
Mortality	7	—	—
SOFA (sequential organ failure assessment)	11.65 ± 5.01	—	—

Data shown are mean ± SD. *P* < 0.05 was considered statistically significant. ^#^One-way ANOVA test; ^†^Pearson chi-square test; ^Δ^Mann–Whitney nonparametric test.

## Data Availability

The dataset generated during this study is available from the corresponding author on reasonable request.
